# PROTOCOL: A systematic review of mobile device use in the primary school classroom and its impact on pupil literacy and numeracy attainment

**DOI:** 10.1002/cl2.1155

**Published:** 2021-04-05

**Authors:** Claire Dorris, Karen Winter, Liam O'Hare, Edda Tandi Lwoga

**Affiliations:** ^1^ School of Social Sciences, Education and Social Work Queen's University Belfast Belfast UK; ^2^ College Of Business Education Dar es Salaam Tanzania

## 1 BACKGROUND

### 1.1 Description of the condition

Children today are born into a world surrounded by technology. For this “i‐Generation” (Twenge, 2017), most aspects of daily life can be conducted online, including socialising, shopping, learning and engaging with the world around them, and the lines between the online and offline life are becoming increasingly blurred. OFCOM, (2019) reports that 82% of 5–7‐year‐olds go online regularly, averaging 9.5 h per week, while for 12–15‐year olds, 99% go online regularly, averaging 20.5 h per week. This increasingly technological world has influenced educational practice, with the use of technology in classrooms evolving from desktop computers, to interactive whiteboards, to the more recent use of tablets and other mobile devices to deliver the school curriculum. A recent development (UNCRC, 2021) has seen the Convention on the Rights of the Child adopt General Comment 25 which recognises children's rights in the digital world. This includes children's right to the educational benefits that technology can bring, and places responsibilities on States to ensure schools are equipped with the infrastructure, knowledge and skills to support this.

Access to technology in the classroom is increasing, however remains disparate across the globe. OECD (2015) reported that in 2012, 72% of 15‐year‐olds in OECD countries use a computer, laptop or tablet at school (unchanged from 2009), although usage in some countries was as low as 50%. The latest OECD (2020) figures report a global average of almost one computer for every 15‐year‐old pupil, with variation between 1.25 computers per pupil in countries such as UK or United States, and 0.25 computers per pupil in countries such as Brazil or Greece. While these figures do not reflect practice in primary‐aged schools, they give some idea of the scale of usage and the potential impact for children and young people around the world. For this reason, the role and impact of technology in the classroom has been a growing area of interest for researchers, spanning topics including the impact on educational outcomes (such as reading, numeracy attainment and critical thinking) (e.g., Bebell & Pedulla, 2015); classroom interaction and pupil motivation to learn (e.g., Campbell & Jane, 2012; Ciampa, 2014) and teacher skills and attitudes to technology (e.g., Ciampa & Gallagher, 2013; Lincoln & Barney, 2017).

As technology has advanced, so too has the range of classroom activities it can be used for. Technology can be used to enhance and support teacher presentation, or can directly engage each child with an individual device or in groups. The integration of technology with pedagogical approaches is therefore of critical importance. Baskerville (2012); Grieffenhagen (2004); Murcia (2014), and others, have considered the ways in which technology can be used to enhance and indeed transform pedagogy, rather than to deliver the same activities with new tools (e.g., a teacher writing a question on a blackboard versus writing it on an interactive whiteboard—the activity has not changed, just the means by which it is presented).

The most recent technological advances, specifically mobile devices, provide an unprecedented opportunity for transformative pedagogy. While tablet‐style computers have been available since the early 2000s, the introduction of the user‐friendly and innovative Apple iPad in 2010, closely followed by similar tablets of other brands, made these tools more accessible to the wider population. Children are particularly skilled in the use of mobile devices from an early age; OFCOM (2019) report that 58% of 3–4‐year olds, and 76% of 5–15‐year olds use a tablet device regularly, while just under half of 5–11‐year olds own one of their own. Additionally, while only 35% of 7–11‐year olds own a smartphone, this figure has been steadily rising (from 24% in 2015). Research by the British Educational Suppliers Association (BESA, 2015) found that 71% of UK primary schools surveyed reported using tablets in the classroom. This was a significant increase from the previous year, with figures predicted to rise significantly by 2020. More recently, and as individual ownership of tablets and indeed smartphones rises, “bring your own device” policies are beginning to be seen across schools to harness the resource now available in pupils' own pockets. The intuitive nature of tablets and smartphones, coupled with affordability and the potential to “bring your own”, make them ideally placed to influence traditional teaching methods. The NMC Horizon Report K‐12 Edition (Johnson et al., 2014) identified such “intuitive technology” as having the potential to significantly impact educational practice over the next 5 years. Children now learn through game‐playing, use the endless body of information available to research topics of interest, and are adept at recording, editing and presenting videos as part of the ordinary school day.

Literacy and numeracy are a central focus of primary education, and not only provide the tools through which a child can engage in wider curricular subjects, but also have far‐reaching application across the life‐course. Indeed, research by the National Literacy Trust (Clark & Teravainen‐Goff, 2018) shows that children who are more engaged with literacy have better mental wellbeing. Yet the National Literacy Trust website also reports that 16.4% of adults in England, 26.7% in Scotland, 17.9% in Northern Ireland and 12% in Wales have “very poor literacy skills”, while OECD (2016) finds that UK young adults (age 16–24) have lower basic literacy and numeracy skills than young adults in many other countries. The 2018 round of PISA tests (OECD, 2019), designed to assess reading, science and maths skills of 15 year‐olds globally, show the UK moving up the rankings in maths (18th, up from 27th in 2016) and in reading (14th, up from 22nd in 2016), yet still falling below many other countries. It is clear that a focus on literacy and numeracy must be a priority for primary age children.

Mobile devices are one way in which teachers in primary schools are enhancing literacy and numeracy education, and there is a general feeling amongst teachers that this is a positive development. The National Literacy Trust (Picton, 2019) surveyed 219 teachers across the UK, and found that just under 60% believe technology can help pupils to overcome learning barriers, and over three quarters feel technology should be available for pupils right across the curriculum to support their literacy development. However despite this belief, trends in technology access and training do not follow. Just under half of the sample of teachers stated that their pupils had access to technology (either laptops or tablets) in the classroom, around 20% of teachers said they never use technology to support literacy, and a quarter reported never having had training to make use of technology to support literacy. Similarly, BESA, 2015 reported that 34% of schools felt their technology implementation was poor, citing an ineffective infrastructure and lack of adequate training and support as barriers. Given the rapid increase in use of mobile devices in the classroom, it is critical that teachers are adequately equipped with the skills, resources and guidance to enable them to effectively and safely embed such mobile technology within pedagogy. Ultimately, this technology must benefit pupils and support learning. The OECD (2015) report also found that those countries reporting heavy investment in technology in schools demonstrated no significant improvement in reading, writing or maths.

While mobile device usage in the classroom continues to grow, its application therefore remains an area of uncertainty, particularly in terms of the impact on pupils' educational experience, and, critically, learning outcomes and attainment. It is important that we can identify what works, for whom, and why. For this reason, this systematic review of existing research on mobile devices in the classroom (specifically tablet devices, smartphones and handheld games consoles), and their impact on attainment, is timely, with an important contribution to make towards ensuring that future developments across educational policy and practice, and in the professional development of educational practitioners, are informed by evidence of good practice.

### 1.2 Description of the intervention

There are three key elements for consideration in how mobile technology is used in the primary school classroom:

1. Devices
2. Activities
3. Outcomes

**Figure 1:**
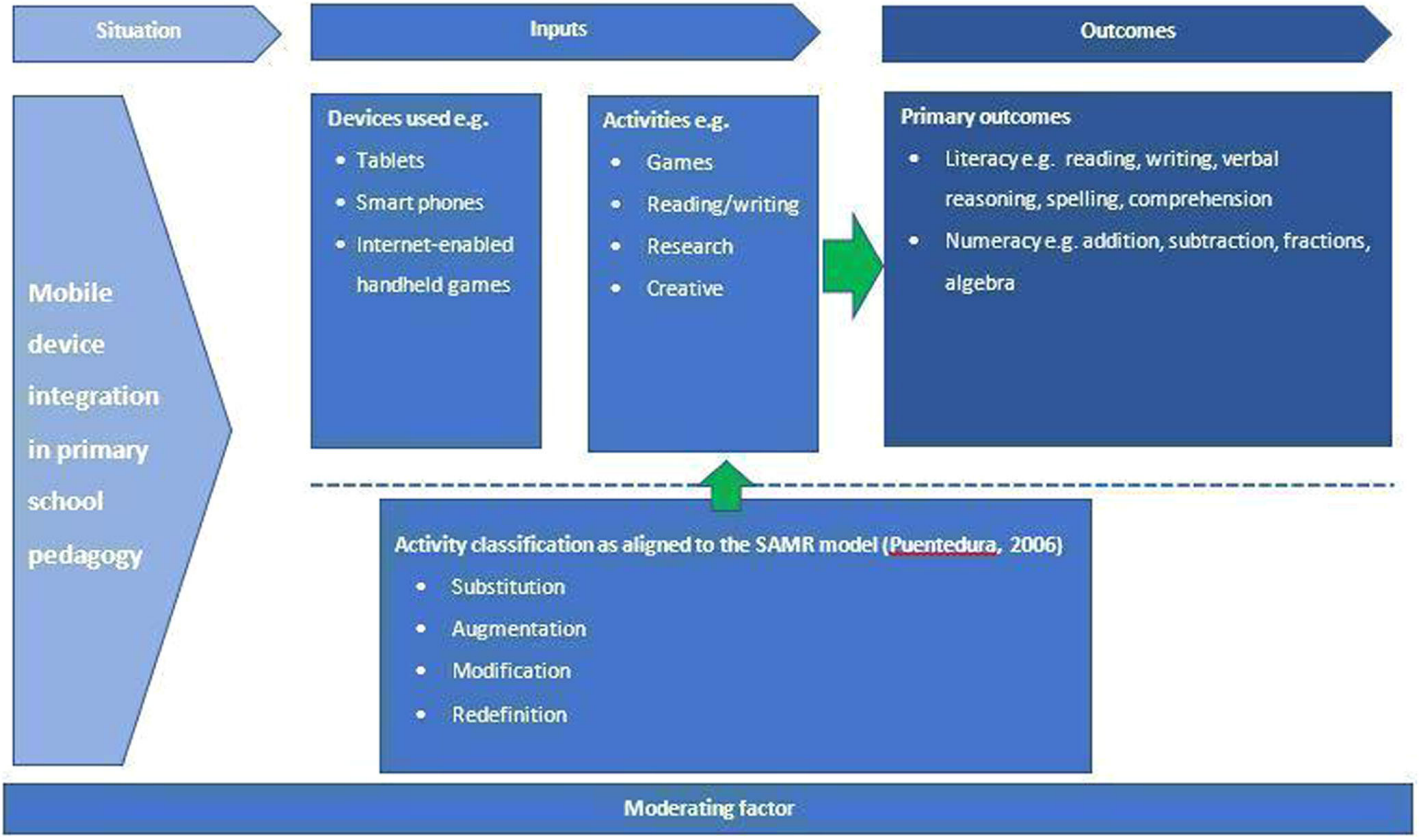
Logic model representing implementation of mobile technology in the classroom

A logic model has been developed to demonstrate how these key components interact (see Figure 1).

#### 1.2.1 Devices

The most common mobile devices used in schools are tablet computers. On first introduction, Apple was the device of choice in US schools, reporting a 95% education market share (Apple, 2013), and while Apple remains the market leader in tablet sales with 36.5% of sales (International Data Corporation, 2020), advances from other brands using Android or Chrome operating systems, and producing less expensive devices, have seen Apple market share in education drop (Futuresource Consulting, 2019). Smartphones are less commonly used in schools; indeed, there is ongoing discussion in the media as to whether children should be permitted to bring such devices to school. In late 2018, the French government passed legislation to ban children and young people (up to age 15) from using their phone in the school grounds during the school day. This move has prompted much debate; those in agreement with this approach feel it addresses concerns such as cyber‐bullying and distraction from studies. Research from the London School of Economics is commonly cited; Beland and Murphy (2015), reviewed exam results following mobile phone bans in schools across England and found not only an improvement in results, but a more significant improvement for those pupils from disadvantaged backgrounds, therefore contributing to a reduction in educational inequalities. Yet the increase in children's ownership of mobile devices undoubtedly presents an opportunity for schools. As ownership of smartphones and tablets increases among children, schools are now considering how they can harness the opportunities presented by children having an Internet‐enabled device in their own pockets (e.g., Rae et al., 2017). Such “Bring your Own Device” approaches also bring challenges, with online safety, appropriate behaviour policies, infrastructure capacity and ensuring equality of access only some of the necessary considerations.

##### Definition of mobile device

For the purposes of this systematic review, the focus will be on mobile devices. These are defined as handheld computing devices, including:

- tablet computers of varying sizes (iPad and other brands)
- smartphones (defined as those with a touch screen interface which can connect to the Internet)
- small handheld games consoles, such as a Nintendo Switch or Nintendo 2DS (again, with a touch screen or integrated buttons and can connect to the Internet or have games loaded).

Within the classroom, the mobile devices will either directly access the Internet, or make use of device applications (“apps”) or inbuilt device functions. While overall, the functionality of these different types of mobile devices will be similar, the screen size of a tablet is likely to be larger than on smartphones or games consoles. Tablets are typically anything between 7 and 10 inches, with some of the more professional models measuring up to 12 inches, while smartphones range in size from 3 to 6.5 inches. There is some evidence that screen size may impact effectiveness, depending on usage. Alghamdi et al. (2014) found that it took longer to read the same information on a small screen than a larger one, however there was no difference in information understanding or retention, while Albó et al. (2019) found there was less opportunity for pupils to collaborate on a small smartphone when used in class, as compared to a larger tablet.

#### 1.2.2 Activities

Mobile devices have applicability right across the curriculum, and can be used in any number of ways, both individually by pupils or in groups. There are also many ways in which the teacher can use mobile devices to support his or her teaching, however these will not be included within the scope of this review. From a brief review of relevant publications, a summary of potential usage and types of activities is included below.

**Table:** 

Aims of activity:	Examples of activities involved	Types of websites or apps accessed
To take in new information or practice new skills	Reading and writing	Word processing software
To communicate with friends, classmates or class teacher	Researching topics of interest Watching instructional videos	Youtube or other video sites
To present information learned	Playing games	Online dictionaries
To demonstrate understanding or knowledge of information	Playing or making music	Specifically designed educational apps and websites, such as: Mathletics, Motion Maths or the Learning Bug Club (details included later in this protocol)
	Drawing pictures or creating art	
	Taking and editing photographs or videos. Undertaking formal tests or informal quizzes	

##### Puentedura's SAMR model (Substitution, Augmentation, Modification, Redefinition)

**Figure 2:**
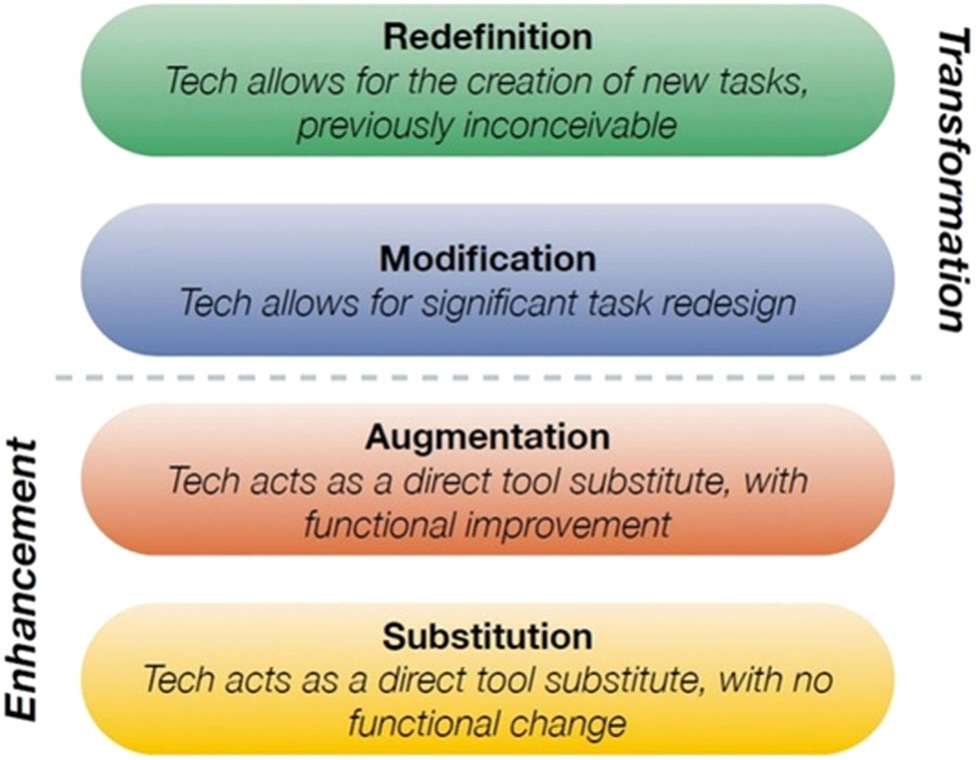
Puentedura's SAMR Model (figure taken from Puentedura's web‐blog)

In any discussion on the impact of mobile device usage in the classroom, the activities undertaken, and the context in which they occur, rather than the device itself, are the important factors. Given the breadth of potential activities, researchers and practitioners have sought a framework to help classify and therefore compare activities. One such model is the SAMR model, developed by Puentedura (2006) (Figure 2). The SAMR model compares activities undertaken using technology with the everyday activities they are replacing (e.g., reading an e‐book rather than a paperback), and asks what the use of technology has added to the learning experience.

Puentedura proposes four levels of activity:

- **Substitution:** Technology is used in substitution for the usual classroom tools or activities, for example, reading an online textbook rather than a paper copy.
- **Augmentation:** Technology substitutes for the usual tools however also improves function slightly, for example, a computer word processor used to write an assignment, therefore allowing for inclusion of pictures or diagrams.

These are both considered to *enhance* pedagogy;

- **Modification:** Technology allows for an activity to be undertaken significantly differently, for example, accessing the Internet to independently research content for an assignment.
- **Redefinition:** Technology allows for new, previously unachievable activities to be undertaken, for example, creating a multimedia assignment using video, audio and other creative tools.

These are both considered to *transform pedagogy*.

Puentedura (2013) emphasises that in practical terms, the model should be seen as a spectrum along which classroom activities sit, and proposes that for true transformation of learning, activities using technology should aspire towards redefinition of pedagogy, rather than simply substituting one activity for another. Geer et al. (2017) also note that as mobile devices were not primarily designed as tools for an educational setting, effort will be needed to adapt them to and embed them within existing pedagogy. The SAMR model has duel purpose. While it contributes to the implementation discussion, it has been criticised for its focus on the technology and activities and ignoring wider modifiers such as teacher knowledge and attitudes, pupil skills and knowledge, and wider dynamics within the classroom (Hamilton et al., 2016). More commonly, SAMR is used as a practice framework to describe and categorise activities, and is increasingly being used to influence good practice and to support teaching professionals in their efforts to transform the pupil experience, informing the types of activities undertaken. A number of teaching websites and resources have sought to provide examples of how the SAMR model can be embedded in practice, and the types of activities that might reflect transformation rather than enhancement. TES, a global online resource for teachers and other educational professionals, highlights the model and the potential benefits in the classroom, and provides a number of resources to support teachers in embedding the approach. To encourage thinking around the model in real terms, Puentedura (2013) proposed a number of questions for educators to ask when introducing new technology or a new activity using technology.

**Table:** 

Substitution:	What is or will be gained by replacing old technology with new?
Substitution to augmentation:	Has an improvement been made to the task process that could not be accomplished with the older technology at a fundamental level?
Augmentation to modification:	Has the original task been modified? How? Does the modification fundamentally depend on the new technology?
Modification to redefinition:	What is the new task? Will any portion of the original task be retained? How is the new task uniquely made possible by the new technology?

Numerous researchers interested in technology use in the classroom have used the SAMR framework to categorise activities taking place (e.g., Fabian & Topping, 2019; Geer et al., 2017). In this systematic review, the SAMR model will be used during the coding stage to classify activities undertaken within interventions in the selected studies, therefore bringing some homogeneity to what is expected to be a diverse range of activities.

#### 1.2.3 Outcomes

Research on the impact of technology in the classroom takes one of two approaches, either focused on the direct impact on pupils' learning outcomes, specifically academic achievement across a range of subjects and measures (primary outcomes), or on the impact that technology has on the teaching environment and pupils' classroom experience, for example, on motivation to learn, engagement, or collaboration with classmates (secondary outcomes). Improvements in secondary outcomes may in turn lead to improvement in primary outcomes. The Education Endowment Foundation (EEF, 2019) reports extensive evidence of “moderate learning gains” when technology is integrated in teaching across a wide range of subjects and age groups, resulting in an additional four months progress on average (EEF “toolkit for teaching and learning” calculations based on impact, cost and strength of evidence). However, EEF conclusions suggest that the type of technology, and the way in which it is integrated within the classroom, vary widely.

*Outcomes of interest in this review*: this review aims to identify and synthesise evidence on the impact that mobile devices have on pupil attainment in terms of literacy and numeracy development, given their wide‐ranging implications for all areas of education and wider life.

##### Defining literacy and numeracy

Early literacy development usually focuses on speaking and listening, before moving onto reading and writing. In supporting children's literacy, there are various components on which the primary school curriculum focuses. The Rose Review (2006), an independent review of the teaching of early reading, carried out on behalf of the Department for Education and Skills, highlighted the need for the systematic teaching of phonics within primary education as a key building block of literacy. This is backed up by a growing body of research, as summarised by the Education Endowment Foundation (2019) Teaching and Learning Toolkit. Other key elements of literacy include vocabulary and spelling, grammar, comprehension (as spelling and grammar knowledge increase), and building fluency, as defined in primary‐level curricula (such as the Northern Ireland Primary Curriculum set by the Council for the Curriculum, Examinations and Assessment (CCEA, 2016); or the National Curriculum for England, set by the Department for Education).

In the same way, curricula for early numeracy education focus on concepts such as basic number recognition, counting, sorting, pattern‐making, weighing and measuring, and these are usually taught through play‐based activities. Once these are mastered, teaching moves on to common mathematical operations such as addition and subtraction, then to application of numeracy skills through such areas as mathematical reasoning, data manipulation and representation. The Northern Ireland Primary Curriculum highlights the need for children's mathematical skills to be relatable to everyday situations and transferable across the curriculum. The elements of literacy and numeracy will be further explored in developing search terms for this review.

A rapid review of a sample of studies (Appendix A) shows that for children in the target population (aged 4–11), researchers have a common interest in improvements in numeracy and literacy attainment. The following research examples demonstrate the range of activities undertaken, outcomes of interest, outcome measures and moderating factors identified:

- Doan and Bloomfield (2014) compared the effects on essay scores of giving children Internet browsing time to research their essay topic before writing. Forty‐nine pupils within a school year were randomly assigned to one of three groups: a control group (business as usual—no Internet browsing time, and 90 min essay‐writing time); intervention group one (30 min Internet browsing time, then 60 min essay‐writing time) or intervention group two (given three by 45 min lessons on Internet search techniques, followed by 30 min independent Internet research time then 60 min essay‐writing time). Essays were scored by independent trained assessors using a scoring rubric developed by the Virginia Department of Education, which assigns a score from 1 to 4 across three domains of composition (ability to express ideas and structure an argument), written expression (tone and “writer's voice”) and usage/mechanics (including punctuation, spelling, sentence structure).
- Hallstedt et al. (2018) used a randomised controlled design to investigate how a maths tablet intervention plus working memory training might affect basic arithmetic for low‐performing pupils (age 8) in the short term. Two hundred and eighty‐three second‐grade pupils across 27 Swedish schools were randomly assigned to one of four groups: control group (no intervention); placebo group (undertook reading activities only); intervention group one (undertook 20 min maths training per day via “Chasing Planets” tablet intervention) and intervention group two (undertook 20 min per day maths training, plus an additional 10 min per day on activities to build their working memory). A combination of standardised tests and national school assessment tests were used, including the Grade Three Math Battery (Fuchs et al., 2003) which measures addition and subtraction fluency; the Heidelberger Recher Test (Haffner et al., 2005) which measures addition, subtraction, missing term recognition and speed; and Diamant AG1 (Swedish National Agency for Education), a Swedish national test which measures addition and subtraction. A number of potential moderating factors were considered in this study, including pupil IQ and socioeconomic status.
- Mak et al. (2017) used a pre and posttest study design to investigate the effects of ABRACADABRA, a web‐based literacy programme, on primary school students in Hong Kong. The programme is aimed at building reading and writing skills in English, either for native English speakers or those for whom English is not their first language. A number of standardised and researcher‐developed measures were used, such as GRADE (Group Reading Assessment and Diagnostic Evaluation), a standardised tool measuring domains such as word reading, listening comprehension and phoneme‐grapheme correspondence (accuracy in reading aloud); and the Literacy Instruction Questionnaire (a teacher questionnaire designed specifically for the programme). The researchers also accessed usage data from each individual pupil over the course of the intervention to see if exposure intensity moderated the findings.

### 1.3 How the intervention might work

The focus of this review is on the specific use of mobile devices in the primary/elementary classroom. Children using the devices will therefore be aged between four and eleven. Given the broad age range, there will be a wide variety in the types of interventions, the complexity and skill needed, and the aims and potential outcomes. The use of the SAMR model to classify the types of activities that might be undertaken and what they can add to existing pedagogy has already been discussed. There have also been a number of theories proposed which can help us to understand how digital interventions might contribute to positive learning outcomes. Some of these are discussed briefly below.

- **Play‐based learning:** Play has a central role in early years and primary education curricula, with a widely established body of research showing the effectiveness of play in learning, from free play through to instructive games. Digital interventions can make learning more fun, creating a positive attitude towards a subject, encouraging creativity (e.g., Livingstone, 2012) and generally enhancing enjoyment of learning (Oliemat et al., 2018).
- **Supporting agency and self‐directed learning:** Geer et al. (2017) report that teachers found tablets to contribute positively to student‐centred learning, with pupils more in control of their learning than traditional teaching could have allowed. This allows students to learn at their own pace, leaving teachers free to provide one to one support where needed and allowing more advanced pupils to move on to more challenging materials.
- **Increasing motivation:** a common focus of research is the role of mobile devices in increasing motivation to learn, which can give pupils a more positive attitude to school work, help them engage more actively in lessons and in turn contribute positively to academic achievement (Ciampa, 2014; Clarke & Abbott, 2015). Tasks associated with increased motivation should provide autonomy, be challenging without being impossible, stimulate the senses, build curiosity, and provide an element of competition, with others or one self (Malone & Lepper, 1987). The use of mobile devices provides opportunity across all these elements.
- **Providing opportunity for formative assessment:** The use of digital devices in the classroom provides additional opportunities for teachers to review and assess pupil progress in real time, and offer feedback on the spot which can support learning (Dalby & Swan, 2019). Many digital interventions are designed with an immediate feedback function which allows the child to see their mistakes and learn from them.

While the content of interventions will vary, the activities (as classified through the SAMR framework) and the characteristics highlighted above, are key factors in how interventions might work, and therefore the potential impact they can have on child outcomes.

#### 1.3.1 Examples of interventions

Some examples of the widely available interventions aimed at primary age children are detailed below.

**Table:** 

Intervention	Academic areas covered	Details of intervention
Motion Math	Maths	Motion Math is an instructional app designed to be accessed via a mobile device, although can also be accessed via a desktop computer. The Motion Math model includes hundreds of levels of mathematics content, aimed at children aged approximately 4–11 and covering general arithmetic concepts aligned to the school curriculum, such as fractions, addition and subtraction, and percentages. Pupils can access the app via their own or school device, log‐in and work through the games and activities at their own pace. Motion Math uses the “tilt” facility on mobile devices so that children physically manipulate the device to engage with activities, for example directing a falling star to the right place. The game facilitates formative assessment, through tracking performance, providing direct feedback to the child, supplying hints and tips if answers are incorrect, and increasing difficulty when answers are correct. Students can work at their own pace through the activities, and teachers receive feedback on pupil usage and performance. A class password is needed to log in.
Top Marks	Maths and literacy	A free website with a range of maths and literacy games for 3–14 year olds. As it is web‐based, it can be accessed via a tablet, smartphone or other Internet‐enabled device, or the app downloaded. Games vary in difficulty, and are broken down into age groupings. Each game provides immediate feedback so the player can try again if they get a question wrong. The website also includes resources for teachers to use as teaching tools via an interactive whiteboard, and can be accessed at home to continue learning for homework.
BBC Bitesize	Maths and literacy	A free website developed and maintained by the BBC (therefore UK‐specific). The site can be accessed at any time via tablet or smartphone, as well as traditional computer, so can be used at home or school. Specific resources are aligned to the curricula across the four nations. A wide range of resources and subject areas are covered for primary, secondary and post‐16 pupils, and combine games, videos and instruction. For the younger users, activities are fun and game based, while for older users, activities make use of “real life” examples.
Mathletics	Maths	Mathletics is a learning platform designed for use in schools and aligned to the UK primary school curriculum, however can also be used at home. Activities can be accessed via tablet or desktop computer, and include a range of tutorials and interactive games. There is a test option available, and pupil activities are marked automatically with detailed reports provided for the teacher. There is also a facility to assign homework. Activities incorporate challenges to motivate individual pupils, with points awarded for completion. Mathletics also includes scheduling and customisation facilities for teachers to support planning.

### 1.4 Why it is important to do this review

The impact that mobile devices actually have on educational outcomes remains unclear. Regardless, investment in tablets for use in the primary school classroom is increasing year on year (BESA, 2015). Given that funding cuts due to wider austerity are commonplace in education as in other areas of life, it is critical that investment is made in the most effective tools and approaches to best support educational outcomes. Educators must therefore be able to access the most up to date evidence to support decision making. A systematic review of existing literature will provide one such accessible resource. The review team has elected to focus on primary education, rather than the full spectrum of educational experience. Primary and post‐primary education, and the use of technology within these, are very different, in terms of the subjects studied, the approach to pedagogy, and activities undertaken. As a team with limited resources, and a desire to produce a review which has the potential to influence practice, we would rather invest in the detail of primary education rather than try to cover both and risk dilution of the discussion.

#### 1.4.1 Existing literature reviews

Within the systematic review discipline, a number of “Coordinating bodies” or “brokerage agencies” (Sundberg, 2009, in Levinson & Prøitz, 2017) have emerged, of which the Campbell Collaboration is one. The primary role of such bodies is to support the review process and provide quality assurance through peer review, so that practitioners, policy makers and others seeking the best available evidence can trust in the robustness of the review. There is currently no existing *registered* systematic review on this specific topic, therefore the team feel it will be an important addition to the robust evidence base.

There are some meta‐analytic studies or literature reviews on a similar theme; these are discussed below.

Haßler et al. (2015) review: “Tablet use in schools: a critical review of the evidence for learning outcomes” is the most similar to the proposed review, however a number of key considerations mean that there remains value in the proposed review:

- This is not a registered systematic review.
- The searches in Haßler et al.'s review were carried out in May/June 2014. The 5 years since the search was carried out has seen rapid growth and evolution of the use of mobile technology in the classroom. Initial literature searches have found a wealth of research since then; the proposed review would draw on this up to date research.
- Haßler et al.'s review considered both primary and secondary school use, did not include smartphones, and focused on wider learning outcomes. There is no subgroup analysis completed, either across age groups or specific learning outcomes, therefore the team feels our proposed review will expand upon Haßler et al.'s findings to better understand primary use specifically. This is particularly important as interventions may impact one group of pupils differently than others, for example younger pupils versus older pupils, or may be effective in maths interventions but not in science. To enable evidence‐informed practice, these details are critical.

There is a current protocol registered with the Campbell Collaboration on a similar theme (title: Free Provision of Information and Communications Technology (ICT) for improving academic achievement and school engagement in students aged 4–18: A systematic review, Liabo et al., 2016). This registered protocol focuses on the impact on academic achievement (including literacy, numeracy and wider knowledge) and on school engagement (as measured by attendance patterns and school enjoyment), of schemes seeking to increase pupils' wider access to ICT, such as discounted laptop schemes or facilitating home Internet access. This ICT is not necessarily for use within the classroom, rather may be used at home or within the community.

A full list of further reviews identified is included in Appendix B along with details of their area of focus and differences in relation to this proposal. This is not a criticism of limitations of existing reviews, rather an attempt to reflect the ways in which the proposed review will add to existing work.

In summary, existing reviews tend to be:

- Focused on older or younger age groups of children (pre‐school, post primary, higher education) without subgroup analysis on our area of interest, therefore while we can get a sense of overall impact of devices, we cannot fully understand how the intervention specifically impacts primary aged children.
- Focused specifically on pupils requiring additional support or with special needs, rather than general usage in the classroom.
- Inclusive of all technologies (including interactive white boards, desktop computers, etc.) rather than focused specifically on mobile devices, or more narrowly focused (e.g., iPad branded tablets only).
- Outdated, particularly given the topic of interest and the rapid evolution of technology applicability in the classroom.
- Reviews or mapping of the types of research carried out in this field, rather than an analysis of the effects of mobile learning interventions.
- Outside of the standards set out by Campbell in terms of systematic review methodology (for example including peer reviewed journals only, or not including grey or unpublished literature).

It is therefore our opinion that a systematic review of research on the specific impact of mobile devices in the primary classroom on literacy and numeracy achievement will build on existing reviews and is of merit and indeed timely.

#### 1.4.2 Policy relevance

Any innovation in the classroom has the capacity to impact all children and young people to a greater or lesser extent; it is therefore critical that educators are equipped with the skills and knowledge to use emerging technology appropriately and effectively to best support pupil attainment. The proposed review has important policy and practice implications across a number of areas, including:

- Curriculum development and delivery;
- Technical provision in schools;
- Teacher training and ongoing professional development;
- Online safety.

## 2 OBJECTIVES

Specifically, the review will aim to answer the following question:

- What is the effect of mobile device integration in the primary school classroom on children's literacy and numeracy attainment outcomes?

This systematic review will focus on the use of tablets (including iPads and other branded devices), smartphones (defined as those with a touchscreen interface and which can connect to the Internet) and handheld games consoles (again with touchscreen interface and Internet connectivity) within the primary school classroom, aimed at improving literacy and/or numeracy for children aged 4–11. The primary objective of this systematic review will be to identify and synthesise high quality research (published and unpublished) to determine the impact of mobile devices in the primary classroom on literacy and numeracy attainment outcomes. Any activities taking place using mobile devices, including apps and Internet sites accessed through them, will be included within the scope of the review

This review aims to support policy makers and practitioners, working in the primary education sector, to make informed decisions about the use of mobile devices in the classroom. The review will therefore seek to address a number of issues within this

- Are there specific devices which are more effective in supporting literacy and numeracy? (tablets, smartphones or handheld games consoles)
- Are there specific activities which are more effective in supporting literacy and numeracy? (aligned to the 4 stages of the SAMR framework—substitution, augmentation, modification, redefinition)
- Do any moderator variables impact on the effectiveness of mobile devices in supporting literacy and numeracy? (specifically gender of user, or intensity of intervention)

Findings from the review will be used to highlight implications for policymakers and practitioners to support their evidence‐informed decision making in relation to the use of mobile devices in primary education. This review also seeks to identify areas where further research is needed in this regard, to ensure that children are getting the best possible support in developing strong literacy and numeracy skills.

### 2.1 Stakeholder engagement

This systematic review will seek to draw available evidence from across the globe, and consider implications for policy and practice which might be relevant to all. Chapter 2 of the Cochrane Handbook (Thomas et al., 2019) highlights the importance of stakeholder engagement throughout the review process, from defining priority topic and review questions through to interpreting review findings in relation to everyday practice. A participatory approach has therefore been incorporated to ensure stakeholder engagement throughout the review process. The involvement of stakeholders in the systematic review process is a less common approach, however the review team feels it is an important inclusion. Cottrell et al. (2015) reviewed studies which considered stakeholder engagement in the systematic review process, to identify benefits and challenges. While the study sample largely reflected medical reviews, the findings are relevant. Benefits and challenges identified include:

- Increased credibility
- Ability to anticipate controversy
- Transparency and accountability
- Improved relevance
- Enhanced quality
- Increased opportunity for dissemination and uptake of findings

Challenges identified include:

- Time required to engage stakeholders
- Training and resources needed
- Engaging appropriate people
- Balancing multiple inputs
- Understanding when and how to engage the stakeholders in the process

The lead reviewer has significant experience of engaging stakeholders in various research and policy development processes, and will use this experience to enhance the review process through the involvement of a small Advisory Group. This group will help to ensure that the review itself, including scope, research questions and interpretation of findings, reflects everyday practice as far possible. The group has already been established, and includes four members representing:

- Primary school teachers (two members, both ICT Coordinators within their schools)
- Education Authority (with significant previous experience at the Council for Curriculum, Examinations and Assessment with responsibility for primary school ICT)
- 1 × Parent (also with significant experience in participatory work with children and young people within a voluntary sector organisation)

To date, the group has been engaged in refining the methodology to ensure this systematic review reflects the current needs & experiences of primary school practitioners as they embed mobile technology in their classrooms. A number of face to face, telephone and email discussions have taken place to inform the direction of the protocol. Initial proposals for the review focused on general technology use in the classroom, including whiteboards, desktop and laptop computers. However, the Advisory Group members advised that while traditional technology has been a feature of classrooms for many years, mobile devices are rapidly becoming a priority for primary schools and bring the potential for more exciting and transformative practice. In their own schools, they have seen significant investment in tablet computers, yet they also noted that the knowledge of colleagues on how to make best use of such devices was limited. The group also discussed “bring your own device” approaches in schools, and while this is not yet commonplace, they felt this was an emerging area of discussion. While mobile devices are being used right across the primary curriculum, literacy and numeracy are areas of commonality across schools, and a significant part of the primary curriculum. The stakeholder group therefore felt that a systematic review of the evidence on mobile device usage to support literacy and numeracy would be of benefit to them and a wide range of colleagues.

Throughout the review, the group will be engaged further to:

- Support the identification of relevant research studies.
- Highlight any relevant groups, activities or events, research, policy or practice developments which may be of interest.
- Support the interpretation of findings of the review in a user‐friendly way which can be understood by a wide range of stakeholder audiences for whom the findings may be relevant
- Support the dissemination of findings to appropriate audiences following completion.

As a voluntary group of expert advisors, time commitment and engagement will be kept to a minimum. Engagement will take place via email where possible, with telephone or face to face meeting only where essential.

## 3 METHODS

### 3.1 Criteria for considering studies for this review

#### 3.1.1 Types of studies

Styles and Torgerson (2018) reflect on the lack of randomised controlled trials (RCTs) in education over the years, highlighting arguments including relevance of the findings to educational practice, the ethics of intervening in pupil learning, and the practical issues involved in randomly assigning interventions when pupils are in fixed classes. However, Connolly et al. (2018) systematically reviewed RCTs in educational research and found that their use has increased significantly and their applicability has been demonstrated. We will therefore seek only studies which report effect sizes for the comparison of an intervention and control group or groups through RCTs (or cluster RCTs). Comparison interventions will include either:

- Traditional teaching methods which do not incorporate technology (no intervention)
- An alternative technology (e.g., desktop computers)

The review will not consider qualitative studies.

#### 3.1.2 Types of participants

The target population will be children within mainstream primary/elementary education settings. Evidence from all countries will be included as long as it meets the wider search criteria. These children will usually be in the age range four to eleven, however on occasion may include children aged twelve.

There may be cases in which both primary and post‐primary aged pupils have been included within a study. In such cases, attempts will be made to isolate the data relating to primary aged children only. If this is not obvious, contact will be made with the author/s to request relevant data; where this is not available, the study will not be excluded, rather will be recorded as having no appropriate data.

Only studies taking place in mainstream schools will be considered. Those which consider use of mobile devices in special schools, educational provision other than at school, or indeed home schooling, will not be included. Additionally, studies which focus on interventions to provide additional support to low‐performing students, rather than the class as a whole, will not be included.

#### 3.1.3 Types of interventions

As already discussed, this systematic review will consider interventions within the classroom in which mobile devices are used to support pupil literacy and numeracy development. Included in the review will be:

- Any intervention or activity within the primary school classroom (with children aged 4–11) that makes use of mobile devices (as defined above) to intentionally support learning in either literacy or numeracy attainment.
- Interventions which are a one‐off or regular activity (however dosage will be taken into account when comparing studies at analysis stage).
- Interventions which engage the class as a whole.
- Interventions where pupils directly use the device, either individually or in pairs or groups.
- Studies focused on specific applications or websites which are accessed through the mobile device will also be included as long as the conditions above are still met.

Excluded will be:

- Interventions which use other technology (such as desktop or laptop computers) rather than mobile devices as specifically defined.
- Interventions which use mobile devices but have no specific focus on literacy or numeracy.
- Interventions where the teacher uses the mobile device to support their own teaching delivery, but pupils have no direct engagement with the device.
- Interventions which do not take place as part of core curriculum delivery (e.g., where pupils take part in activities during free time).
- Interventions which do not take place within mainstream classroom (for example where homework is set via activities on mobile devices, or where the device is used in an after school group).
- Interventions targeted at children with learning difficulties or delays.

#### 3.1.4 Types of outcome measures

##### Primary outcomes

This review will focus on primary outcomes, that is, interventions which have a direct impact on children's academic achievement in literacy and numeracy. A wide range of measurement methods are employed to assess outcomes across literacy and numeracy, including both standardised, national assessments and bespoke tools, usually quantitative but sometimes observational in nature. The core academic assessment of the country (such as key stage 1 or 2 in the UK or Children's Progress Academic Assessment [CPAA] in the United States) is also commonly used where scores are allocated to individual pupils. For the purposes of this systematic review, studies which focus on improvement in any element of literacy or numeracy will be considered for inclusion. However, specific outcome measures will not be used as a criteria for study inclusion or exclusion.

Some common elements of literacy and numeracy have already been discussed. These will be further distilled in developing search strings for information retrieval.

##### Secondary outcomes

While the literature on technology integration in primary school classrooms considers a wide range of secondary outcomes, including enhanced motivation and engagement with peers (e.g., Ciampa, 2014), this systematic review will focus only on primary outcomes relating to academic attainment.

### 3.2 Search methods for identification of studies

The following section describes the proposed methodology for identifying studies for inclusion. The methodology is designed to minimise the risk of publication bias, ensuring that all relevant studies are captured.

#### 3.2.1 Electronic searches

##### Search limitations

**Year of publication:** while the first commercially available touch‐screen smartphones were introduced in the mid 2000s, and tablets followed towards the end of the decade, there were a number of Personal Digital Assistants available in the 1990s, for example the Delaware Fingerworks devices (later bought over by Apple), Palm Pilot or the Apple eMate, which may have been used in education. It is important that any research on these early devices is captured in the searches to enable a reflection on advancement in technology and approaches, therefore it is reasonable to limit searches to1990 onwards, given that the technology of interest was not available before then.

**Language:** Language will not be used as a search limiter, as this would leave the review open to bias. Effort will be made to obtain an English translation of any study thought to have relevance, using available online translation tools or contact with the author/s. Where a translation cannot be obtained, the study will be included as “awaiting classification” and the potential for bias in this regard will be reflected upon in the final report.

**Geography:** No limitations will be placed on geographical location of the study.

###### Search terms

Search terms will be grouped as follows:

1. **Population of interest** (combining broad terms for appropriate age with class/classroom/school) OR
2. **Setting:** mainstream primary school setting, or global equivalents
3. **Intervention of interest:** (a) type of mobile device used (tablets, smartphones, handheld games consoles; all touchscreen and internet‐enabled) and (b) curricular topic addressed (i. literacy OR ii. numeracy and associated concepts)
4. **Study design:** randomised controlled trials

The search strategy has been designed to deliver a more comprehensive, or “sensitive”, search. While this increases the risk of irrelevant studies being identified, these will be screened out through abstract review (discussed in the next section). Search strings will be combined as follows:

###### (1 OR 2) AND (3a AND (3bi OR 3bii)) AND 4

An initial list of search terms within each grouping has been drawn up using a combination of keywords (Table 1) and a sample search string developed by combining each grouping within ERIC (Appendix C). This was developed by reviewing keywords from a sample of randomly selected, relevant articles, as well as reviewing the subject terms used in ERIC and British Education Abstracts. As databases vary, final search terms will be tailored to suit each through a review of the database thesaurus. Where available, database limiter functions will be used for setting (education level), rather than inputting a search string. For Google Scholar search, a smaller, more targeted search string will be used given the search function is limited to 256 characters (including operators). A record of each search completed will be kept, including date of search, specific combination of keywords used, total numbers of studies identified and retrieved.

**Table 1:** Sample search terms

	Concept 1	Concept 2	Concept 3a	Concept 3bi	Concept 3bii	Concept 4
Key concepts	Population: children aged 4–11	Setting: primary school or equivalent	Intervention: Mobile device	Intervention: Subject	Intervention: subject	Study design
Free text terms/natural language terms	Age 4, 5… (up to 11) 4 year old… (up to Child Boy Girl AND school class Classroom	Primary school Elementary school Junior school Kindergarten Grade 1, grade 2, and so forth. 1st grade, 2nd grade, and so forth. First grade, second grade, and so forth	iPad tablet Tablet computer* touchscreen Handheld device Portable computer e‐book OR ebook e‐reader OR ereader electronic storybook Game* console Digital game Smartphone smart phone Mobile phoneiPhone Cell* phone Portable cell* phone Mobile telephone Cell* telephone Personal Digital Assistant PDA* Transportable Cell* Phone 1:1 computing Online instruction M‐learning	Literacy Reading Writing Handwriting Transcription Verbal reasoning Critical thinking Comprehension Notetaking Composition Listening skills Phonics Phonemic awareness Alphabet Spell* Vocabulary Punctuation Grammar Reading fluency Speaking skills Spoken language Critical literacy Literacy skills	numeracy number* math* arithmetic sums calculat* addition adding subtract* multiplication division count* algebra fractions decimal* geometr* statistic* “problem solving” “shape sort*” “mathematical literacy” “quantitative literacy”	RCT Randomised Control Trial Randomised Controlled Trial Randomized Control Trial Randomized controlled Trial Placebo Random allocation Random assignment single/double/triple blind
Subject terms/headings and/or controlled vocabulary (from British Education index)		Elementary education Elementary schools Elementary education research Primary education Primary schools First grade (education) **[repeat for all grades]** School children Elementary school teaching Primary school teaching Computers in elementary education	Educational technology Computers in Education Educational innovations Internet in Education Computer Assisted Instruction Digital Technology Education‐Computer applications Computer games Educational games Handheld devices Technology integration	Reading Achievement Literacy Education Reading comprehension Reading achievement English language education in primary schools Computers and literacy Literacy research Literacy program*Reading (elementary) Reading (primary) Reading enrichment Reading mobile apps	Mathematics achievement Numerical calculations Mathematics instruction Mathematics education (primary) Mathematics education (elementary) Mathematics education (primary) Mathematics education (preschool) Games in mathematics education Study and teaching of numeracy Mathematical ability	RCT Randomised Control Trial Randomised Controlled Trial Randomized Control Trial Randomized controlled Trial
Free text search strings (tested through ERIC on EBSCO)	AB (“Age* 4" OR “age* 5" OR “age* 6” OR “age* 7” or “age* 8” OR “age* 9” OR “age* 10” OR “age* 11” OR “4 year* old*” OR “5 year* old*” OR “6 year* old*” OR “7 year* old*” OR “8 year* old*” OR “9 year* old*” OR “10 year* old*” OR “11 year* old*” OR Child* OR boy OR girl) AND (school OR class* OR classroom)	AB “Primary school” OR “Elementary school” OR “Junior school” OR Kindergarten OR “grade 1” OR “grade 2” OR “grade 3” OR “grade 4” OR “grade 5” OR “First Grade” OR “Second Grade” OR “Third Grade” OR “Fourth Grade” OR “Fifth Grade” OR “1st grade” OR “2nd grade” OR “3rd Grade” OR “4th Grade” OR “5th grade“	AB iPad OR tablet OR “tablet computer*” OR touchscreen OR app OR “handheld device” OR “handheld computer” OR “PDA” OR “personal digital assistant” OR “portable computer” OR '"e‐book” OR ebook OR “e‐reader” OR ereader OR “electronic storybook” OR “game* console” OR “digital game” OR smartphone OR “smart phone” OR “mobile phone” OR iPhone OR “cell* phone” OR “portable cell* phone” OR “mobile telephone” OR “cell* telephone” OR “transportable Cell* Phone” OR “1:1 comput*” OR “online instruction” OR “mobile learn*” OR M‐learning	AB Literacy OR Reading OR Writing OR Handwriting OR Transcription OR “Verbal reasoning” OR “Critical thinking” OR Comprehension OR Notetaking OR Composition OR “Listening skills” OR Phonic* OR “phonemic awareness” OR Alphabet OR Spell* OR Vocabulary OR Punctuation OR Grammar OR “Reading fluency” OR “Speaking skills” OR “Spoken language” OR “Critical literacy” OR “literacy skills"	AB numeracy OR number* OR math* OR arithmetic OR sums OR calculat* OR addition OR adding OR subtract* OR multiplication OR division OR count* OR algebra OR fractions OR decimal* OR geometr* OR statistic* OR “problem solving” OR “shape sort*” OR “mathematical literacy” OR “quantitative literacy”	AB RCT OR “randomised control trial” OR “randomised controlled trial” OR “randomized control trial” OR “randomized controlled trial” OR randomised OR randomized OR placebo* OR (random* AND (allocat* OR assign*)OR (blind* AND (single OR double OR treble OR triple))

##### Search sources

The search will incorporate relevant journal and other databases, accessed through the QUB library, with a particular focus on education. As recommended by *Campbell Method Guide 1: Searching for studies* (Kugley et al., 2016), both field‐specific and multi‐disciplinary databases will be searched. We aim to retrieve published and unpublished studies, journal and non‐journal studies (including NGO and government research), conference papers and reports on proceedings, technical reports, dissertations and theses, white papers and other relevant literature. The following electronic databases will be prioritised:

**Journal databases (and interface through which they will be accessed—if applicable):**

- British Education Index (EBSCOhost)
- Child Development & Adolescent Studies (EBSCOhost)
- Cochrane Central Register of Controlled Trials
- Directory of Open Access Journals (DOAJ)
- Education Abstracts (EBSCOhost)
- ERIC (Education Resources Information Centre) (EBSCOhost)
- International Bibliography of the Social Sciences (IBSS) (ProQuest)
- Education Journals (ProQuest)
- PsychInfo (OVID)
- Scientific Electronic Library Online (SciELO)
- Scopus
- Social Science Citation Index (Web of Science)

**Review databases:**

- Campbell Collaboration
- Cochrane Database of Systematic Reviews
- EPPI Centre Database of Education Research

**Other relevant databases:**

- OECD Education iLibrary
- Current Educational Research in the UK (CERUK)
- EducationLine (EBSCOhost)

**Unpublished studies:**

All efforts will be made to ensure that unpublished studies are identified. To do this, searches will be conducted through:

- Opengrey
- Google Scholar—the first 500 hits will be screened for relevance. Google activity controls will be used to turn off search history, location services and other personalisation options to ensure this does not impact search results.
- Microsoft Academic Search
- ProQuest Dissertation and Theses
- Government websites (limited to those available in English)
- European Documentation Centre
- Websites of charities and funding organisations (including Education Endowment Foundation, National Literacy Trust, National Numeracy Trust, British Educational Research Association)

Again, search statements will be modified to suit the source; advanced search options will be used where available.

#### 3.2.2 Searching other resources

Alongside the main electronic searches, a number of other activities will take place to ensure inclusion of all eligible studies.

- Contact will be made with authors prominent in the subject area (first and second authors of included studies, plus any others who have appeared regularly in excluded but relevant studies) to identify any unpublished studies or work in progress, either of their own or known to them.
- The authors have identified the British Journal for Educational Technology, and Computers & Education as the two most relevant journals, based on a triangulation of journal metrics and expert experience. The most recent editions of each will be retrieved and hand‐searched for studies which meet the inclusion criteria.
- Alongside any conference proceedings identified through the grey literature searches above, authors have identified the following conference/s as being highly relevant: International Society for Technology in Education; BETT; British Educational Research Conference and the European Conference on Education. These have been selected given their global reach, relevance to primary education and technology, and focus on research and pedagogy rather than marketing opportunities for technological products. The conference proceedings from the past 5 years will be searched by hand to identify those not yet indexed in the commercial databases.
- Reference lists of included studies will be reviewed, relevant studies identified and articles retrieved online (via QUB database). Bibliographies of other relevant systematic reviews or meta‐analyses will also be reviewed and relevant studies identified and retrieved.
- A citation index search will also be carried out through relevant databases to identify any more recent studies which have cited already identified studies.
- Where a potentially relevant study has been identified however is not available online, the author/s will be contacted via email to request a copy.

All searches will be fully documented to the degree that the searches will be fully replicable.

### 3.3 Data collection and analysis

#### 3.3.1 Selection of studies

All initial searches will be carried out by the lead author (C. D.), following the strategy set out above. Eligible studies will be imported into EPPI‐Reviewer and duplicates identified and removed before screening. A number of trial searches have already been run and the number of returns is not expected to be high; this is largely due to the inclusion of an RCT filter, which significantly narrows down the potential number of studies for inclusion.

The review is being carried out as a Doctoral Dissertation with supervisors as supporting co‐authors, therefore there is limited capacity within the team. Should the search return 1000 records or less, dual screening of all records will take place at title and abstract stage. The following process will be followed to ensure robustness of the process. A test batch of the same 50 records will be allocated to all four reviewers for screening, then Cohen's *κ* coefficient (*k*) calculated to measure inter‐rater reliability across these records. This process will be repeated, allocating further batches of 50 records for screening by the full team, until satisfied that decisions are consistent across the team and that the screening questions are appropriate. As per Cohen's original discussion, a *k* value of 0.41 or greater will be considered acceptable. Once satisfied with decision making, the lead author will screen all remaining records, and in addition, distribute them among co‐authors (K. W., L. O.'H., T. L.) for independent screening, meaning that all records receive dual screening. Should more than 1000 records be returned from the search process, the lead author will screen all records, and distribute a random sample among co‐authors to ensure 1000 records have been independently screened by two authors.

Full text of remaining studies deemed relevant, or where it is unclear as to relevance, will then be retrieved and each record will be screened in duplicate at this stage. The lead author will screen all records, and will distribute all records among the three co‐authors to ensure each record is screened by two independent reviewers.

The following screening questions are proposed:

1. Does the study consider use of mobile devices in the classroom?
2. Are study participants in the correct age group (4–11) and within a primary school (or equivalent) class setting?
3. Is the intervention aimed at improving literacy and/or numeracy and related skills?
4. Do pupils use the device themselves (rather than the teacher)?
5. Is an RCT used?

Where the answer is no to any one of the above questions, the study will be eliminated and no further questions need be answered.

Throughout the process, details of any studies identified which have not yet been completed or reported on will be recorded and revisited before publication. The screening process will also be fully documented using a PRISMA Flow Diagram as specified in chapter 4 of the Cochrane Handbook (Lefebvre et al., 2019). A list of 'characteristics of excluded studies' will be compiled for those studies which appear to meet the eligibility criteria but have been excluded for a specific reason.

#### 3.3.2 Data extraction and management

When the final list of eligible studies has been identified, coding will take place. A coding framework (see Appendix D) has been adapted from guidelines set out in “Chapter 5: Collecting data” from the Cochrane Handbook (Li et al., 2019). This will be refined following identification and review of the final studies. As the team has limited resources, it is not anticipated that all studies will be dual coded. Instead, all studies will be coded by the lead reviewer, while a 20% sample will be independently coded by a second reviewer. Again where possible, Cohen's k will be calculated to measure inter‐rater reliability, and if not satisfactory (*k* = 0.41 or greater), a further sample of 20% will be independently double coded. Throughout the search, screening and coding processes, any disagreements will be resolved through discussion with all reviewers until consensus is reached.

Coding will focus on the following information:

- Study identifiers and background information (e.g., authors, geography, year, ID, source)
- Characteristics of sample/participants (e.g., age, gender, country, ethnicity, sample size, demographics)
- Intervention details (such as setting information, location, type of device, activity, frequency, outcome of interest, delivery approach, assessment of SAMR classification (substitution, augmentation, modification, redefinition)). The Template for Intervention Description and Replication (TIDieR) (Hoffman et al., 2014) will be used to guide the information recorded.
- Study design (e.g., specific outcomes measured, tools or instruments used, methods of data collection, timing of data collection, effect sizes)

Where the population studied includes children outside of the specified age group (4–11), contact will be made with the author/s to determine if disaggregated data is available. If not, the study will be considered for inclusion if the majority of the study population is within the specified age group. If this is the case, the review team will discuss the implications for the study, consult with the Expert Advisory Group, and undertake sensitivity analysis (discussed below) to assess potential impact on findings, ensuring they “keep faith with the objectives of the review” (Cochrane Handbook, section 3, McKenzie et al., 2019). Where decisions such as these have been made, justification will be fully documented in the final review.

#### 3.3.3 Assessment of risk of bias in included studies

As this review will only include randomised studies, the latest version of the Cochrane “Risk of Bias” (RoB2) tool will be used to assess for bias in individually randomised trials, including the variant tool for cluster‐randomised trials (Sterne et al., 2019). Studies will be rated as low, high or unclear risk of bias. Risk of bias will be independently rated by two reviewers, and disagreements resolved in discussion with a third reviewer.

#### 3.3.4 Measures of treatment effect

Summary data will be collected from each included study, and meta‐analysis will be carried out if sufficient and appropriate studies are identified. It is assumed that there will be variability across the studies, for example in population or implementation of interventions, as well as through sampling error, therefore a random effects model will be used throughout. While the outcomes of interest (literacy and numeracy) have been stated, the ways in which these have been measured will differ across studies. Results will therefore be standardised to allow comparison. Where the dependent variable is continuous, standardised mean difference (*d*) or correlation coefficient (*r*) will be calculated, and where there is a dichotomous dependent variable, odds ratio will be calculated. Primary information to be collected will include mean value of the outcome measure, standard deviation for each intervention group, and number of participants in each group.

#### 3.3.5 Unit of analysis issues

Studies, rather than reports of studies, will be the desired unit of analysis, however the search may return a number of reports of various aspects of the same study. During the screening process, the review team will manually identify and link multiple reports of the same study to avoid “double counting”. All reports of the same study will be reviewed to determine the most appropriate information for inclusion. Any additional reports of the study will be used to glean background information.

Should any study have two intervention groups with one control group, the intervention groups will be combined (if similar), otherwise the Robust Variance Estimation (RVE) method will be adopted to deal with nonindependent effect sizes (Hedges et al., 2010). Similarly, RVE will also be used to perform meta‐regression where there are multiple outcome measures reported for the same outcome domain (correlated effects) within a single study.

Where studies report multiple outcome measures for the same construct at different follow up periods, the main meta‐analysis will focus on outcomes measures immediately posttest. Any follow up data will be grouped into similar time periods and separate meta‐analyses carried out. Consideration will also be given to the intensity/dosage of intervention, with care taken to compare only studies with similar intensity.

Given that the included studies will be set in schools, it is likely that cluster randomised trials will be included. In this case, the unit of allocation will be a group or cluster. In such cases, we will assess whether the study has been appropriately adjusted for clustering (for example through the use of multi‐level modelling), then follow procedures to estimate effective sample size using an estimate of intracluster correlation coefficient (ICC) as outlined in the Cochrane Handbook Chapter 23 (Higgins et al., 2019).

The review team will discuss and document any decisions made in selecting the primary data for inclusion.

#### 3.3.6 Dealing with missing data

Where the study report is missing key data, the reviewers will attempt to calculate the required measures from reported data (e.g., calculating standard error from confidence intervals or *p* value). However, if this is not possible, the author will be contacted to request data. Should the data not be accessible the study will not be included in the meta‐analysis (however will still be reported).

#### 3.3.7 Assessment of heterogeneity

Heterogeneity refers to variation in effect sizes across studies. Cochran's *Q* will be calculated to assess whether any differences between studies are due to chance alone. As *Q* has low power when the number of studies is low (as expected in this meta‐analysis), I^2^ will also be calculated and reported, with *I* > 50% considered moderate heterogeneity and *I* > 75% considered large heterogeneity.

#### 3.3.8 Assessment of reporting biases

A number of different reporting biases will be assessed throughout the review process, including:

Publication and time‐lag bias: The search strategy reported above is constructed to minimise risk of publication bias, including multiple publication, or nonpublication. A funnel‐plot will also be constructed (study precision against effect size) and inspected for symmetry, however if the number of studies is low, bias may remain unclear. If appropriate, Egger's regression test may also be used (Egger et al., 1997).

Outcome reporting bias: There may also be bias in terms of the specific outcomes reported on in a study, with data only partially reported, particularly if one or more outcome areas or subsets produce more significant findings. As above, the RoB2 tool will be used to assess potential bias in this regard.

Location and language bias: language and location will not be used to limit searches, and translations will be sought where studies are not presented in English. As noted above, where a translation is not available, the study will be included as 'unclassified' and potential bias assessed and discussed (see sensitivity analysis below).

#### 3.3.9 Data synthesis

If there are two or more studies with common characteristics which can be meaningfully and logically grouped together, meta‐analysis will be carried out. Rev Man will be used to synthesise the main effects across all identified studies, and for each outcome area (literacy and numeracy). This will include weighted mean effect size, standard error and confidence interval. Forest plots will be used to display findings. In the event that there are not sufficient studies to undertake meta‐analysis, a narrative synthesis will be undertaken and reported.

#### 3.3.10 Subgroup analysis and investigation of heterogeneity

Again where meaningful we will undertake subgroup analysis to identify any specific characteristics that may have a greater or lesser effect; this will be done using meta‐analytic regression on the following moderating factors:

- Activity classification via the SAMR framework; that is, activities are judged to reflect substitution, augmentation, modification or redefinition as per Puentedura's definitions.
- Screen size: as discussed earlier, research suggests a number of ways in which screen size may impact usage and outcomes, therefore it will be important to determine if this is a moderating factor. This will have important implications for future practice, particularly since the number of children with their own smartphone (with typically smaller screens than tablets) is increasing (OFCOM, 2019).
- Gender: In the early days of computers, boys were considered to be more enthusiastic users (Bergin et al., 1993). Recent research concludes that girls and boys now spend similar amounts of time using technology, and are equally proficient, however their activities differ, with girls more likely to use computers for homework or social media, while boys are much more likely to play computer games (Mullan, 2018). Any potential difference in impact across the genders in terms of educational outcomes will have implications for practice.
- Intervention frequency: defined as low, medium or high (these will be more clearly defined by reviewing the included studies following coding).

#### 3.3.11 Sensitivity analysis

Although effort will be made throughout the review process to remain objective, there are various stages at which decisions made may impact final conclusions (decision nodes), as per Cochrane Handbook Chapter 10 (Deeks et al., 2019). Sensitivity analyses will therefore be undertaken to demonstrate that the review is robust despite any decisions made or eligibility criteria employed: an example is the discussion above where a sample may include some children outside of the specified age range. A subjective decision will be required from the review team as to inclusion.

Sensitivity analysis will also be conducted to determine if any of the following unduly influence the findings:

- Studies with a high or unclear risk of bias
- Studies with incomplete data
- Studies with outlier effect sizes (identified through a funnel plot)

All decisions will be fully documented in a summary table, and steps taken to resolve any issues that may adversely impact the strength of conclusions drawn.

## CONTRIBUTIONS OF AUTHORS

This systematic review and meta‐analysis will form the basis of Claire Dorris's Doctoral Dissertation, being undertaken via the DChild course at the School of Social Sciences, Education and Social Work at Queen's University, Belfast. Claire will be primarily responsible for all aspects of the review, with guidance from her supervisors, Prof. Karen Winter and Dr. Liam O'Hare, and from Prof. Edda Tandi Lwoga at the University of Tanzania, all co‐authors on this review. Prof. Winter and Dr. O'Hare will advise on the wider review process including information retrieval, outcome measures and statistical analysis. Prof. Lwoga will advise and support in developing the search strategy and in information retrieval.

## DECLARATIONS OF INTEREST

There are no conflicts of interest noted for any of the authors.

## APPENDIX A A RAPID REVIEW OF OUTCOME MEASURES IN A SAMPLE OF STUDIES

**Table:**

Reference	Domains measured	Tool/s used
Hallstedt et al. (2018) Short and long‐term effects of a mathematics tablet intervention for low performing second graders. *Journal of Educational Psychology*, 2017, vol. 110 (8), pp. 1127–1148.	Arithmetic Word problem solving Cognitive	Arithmetic: · 4 Grade 3 Math Battery scales · HRT · Arithmetic Composite · Diamant AG1 Word problem solving: · Klurisen, measuring problem solving · Word Problem Solving Test by Jitendra (Jitendra et al., 2007) Cognitive: · Raven's Standard Progressive Matrices (Ravens). · Grid and Digit‐Span Backwards.
Bebell and Pedulla (2015) A quantitative investigation into the impacts of 1:1 iPads on early learner's ELA and math achievement. *Journal of Information Technology Education: Innovations in Practice*, 14, 191–215.	English Language Arts Maths	ELA: Rigby Benchmark Assessment Children's Progress (2009) Academic Assessment (CPAA—standard United States academic assessment)· Phonics/Writing Test (PW) · Test (List) · Phonemic Awareness Test (PA) · Reading Test (Read) Observation Survey of Early Literacy Achievement (OSELA) · Letter Identification (LetID) · Concepts about Print (CAP) · Word Reading/Ohio Word Test (OWR)· Writing Vocabulary (WV) · Hearing and Recording Sounds in Words (HRSIW) · Text Reading
Bouta and Retalis (2013) Enhancing primary school children collaborative learning experiences in maths via a 3D virtual environment Education and Information Technologies (2013) vol. 18: 571	Engagement in a task	Measured through chat log files within specific computer programme Observation—video recording then coding
Clarke and Abbott (2015) Young pupils', their teacher's and classroom assistants' experiences of iPads in a Northern Ireland school: “Four and five years old, who would have thought they could do that?” *British Journal of Educational Technology*, 2015, Vol. 47(6), pp.1051–1064.	Motivation Confidence Collaboration Listening skills Fine motor skills	Observation and interviews (via circle time)
D'Agostino et al. (2015) Introducing an iPad app into literacy instruction for struggling readers: Teacher perceptions and student outcomes. *Journal of Early Childhood Literacy*, 2015, vol 16(4), pp.522–548.	Literacy	Observation Survey of Early Literacy Achievement (OSELA)
Gustad (2014) The impact of technology tools on literacy motivation on elementary school English language learners: podcasting in a 4th grade EAL class. *The International Schools Journal*, 2014, vol. 34(1), pp.75–84.	Motivation to read	Motivation to Read Profile (MRP) reading survey
Jesson et al. (2015) Raising literacy levels using digital learning: a design‐based approach in New Zealand. *The Curriculum Journal*, 2016, Vol 26(2), pp. 198–223.	Literacy	e‐asTTle assessment tool. This is a standardised online assessment, based on the New Zealand Curriculum (Ministry of Education, 2007
Kiger et al. (2012) Examining the Influence of a Mobile Learning Intervention on Third Grade Math Achievement. Educational Technology, Research & Development, 2012, Vol. 45(1), pp.61–82.	Maths	Bespoke post‐intervention multiplication test, administered under standardised conditions.
Lee (2017) Learning vocabulary through e‐book reading of young children with various reading abilities. *Reading and Writing*, 2017, vol. 30(7). Pp.1595–1616.	Vocabulary	The TOSREC—standardised reading test, measures speed & accuracy of silent reading Bespoke vocabulary test
Liu (2012) Effects of Integrating Multimedia into the Third Grade Mathematics Curriculum to Improve Student Learning. *Journal of Educational Technology Systems*, 2012, vol. 40(3), pp.251–271.	Maths	Bespoke pre/post‐test developed Bespoke student feedback survey (Likert scale, e.g., how did you like the content of the lesson)
Looi et al. (2015) Exploring Students' Progression in an Inquiry Science Curriculum Enabled by Mobile Learning. *IEEE Transactions on Learning Technology*, 2015, vol. 8(1), pp.43–54.	Science	Standard academic assessment (this example is Singapore)
Miller (2011) Educational benefits of using game consoles in a primary classroom: A randomised controlled trial. *British Journal of Educational Technology*, 2010, Vol. 41(2), pp.242–255.	Mathematics—performance and attitude	100 item “number challenge” (Miller & Robertson 2009) Marsh's Self‐Description Questionnaire (measures self‐perception)
Moon et al. (2017) Enhancing Reading Comprehension with Student‐Centered iPad Applications. TechTrends, 2017, Vol. 61(2), pp.187–194.	Reading comprehension	Bespoke reading habits survey (Likert) AIMSweb Reading comprehension assessment (MAZE)
Musti‐Rao & Plati (2015) Comparing Two Classwide Interventions: Implications of Using Technology for Increasing Multiplication Fact Fluency. *Journal of Behavioural Education*, 2015, vol. 24(4), pp.418–437.	Maths	App immediate score feedback
Outhwaite et al. (2017) Closing the gap: Efficacy of a tablet intervention to support the development of early mathematical skills in UK primary school children. *Computers & Education*, 2017, vol. 108, pp. 43–58.	Maths	Bespoke tools based on Numerical Operations sub‐test of the WIAT‐II (Wechsler, 2005). Bespoke 50 item quiz measuring curriculum knowledge.
Sessions et al. (2016) The Neglected “R”: Improving Writing Instruction Through iPad Apps. *TechTrends*, 2016, Vol. 60(3), pp.218–225	Student writing skills Motivation Engagement Classroom dynamics	Developmental Reading Assessment Analysis of daily learning journals Semi‐structured interviews
Yinikoglu & Gogus (2017) Use of handwriting recognition technologies in tablet‐based learning modules for first grade education. *Educational Technology Research & Development*, 2017, Vol. 65(5), pp.1369–1388.	Motivation to write	Bespoke questionnaire developed (pre and post‐intervention) Observation
Savage et al. (2013) A (Pan‐Canadian) cluster randomized control effectiveness trial of the ABRACADABRA web‐based literacy program. *Journal of Educational Psychology*, 105(2), 310–328. https://doi.org/10.1037/a0031025	Literacy	Comprehensive Test of Phonological Processing (CTOPP) Fry's Instant Word List (Fry et al. 2000) Group Reading Assessment and Diagnostic Evaluation (GRADE) Dynamic Indicators of Basic Literacy Skills (DIBELS; Kaminski & Good, 1996) Subtests: Oral Reading Fluency (ORF) & Phonemic Segmentation Fluency subtest

## APPENDIX B SUMMARY OF EXISTING SYSTEMATIC REVIEWS AND/OR META‐ANALYSES ON SIMILAR THEMES

**Table:**

Author	Title	Year	Population	Type of technology	Scope of review	How does the review differ from the proposed review?
Haßler et al. (2015)	Tablet use in schools: a critical review of the evidence for learning outcomes.	2015	5–18	Tablet	Academic achievement across any learning outcome. Included: literature, maths, social studies, science, economics, SEN	This is not a registered systematic review Searches were completed May 2014. Initial scoping of research shows a significant growth in research since then. Anecdotal evidence suggests that practice has shifted significantly since 2014 too in terms of iPad/tablet use in schools, given the first tablet was introduced in 2009 (android, 2010 iPad). This review considered both primary and post‐primary schools, did not include smartphones, and focused on wider learning outcomes. There is no subgroup analysis completed, either across age groups or specific learning outcomes, therefore the team feels our proposed review will expand upon Haßler et al.'s findings to better understand primary use specifically
Abrami et al. (2015)	The effects of ABRACADABRA on Reading outcomes: A meta‐analysis of applied field research	2015	Kindergarden and elementary or equivalent (5–12)	Abracadabra (interactive, web‐based software for reading support)	Reading outcomes	Meta‐analysis on research on a specific intervention (Abracadabra)—the scope of the proposed review will consider literacy and numeracy interventions more widely
Cheung and Slavin (2012)	The Effectiveness of Educational Technology Applications for Enhancing Mathematics Achievement in K‐12 Classrooms: A Meta‐Analysis. Best Evidence Encyclopaedia (BEE)	2012	Grades K‐12 or equivalent (5–18)	Wider technology	Impact on maths achievement where pupil was previously struggling	This meta‐analysis focused on support for pupils who are struggling or require additional support, rather than on general application in the classroom (which the proposed review will consider). Searches identified studies only up to 2011. Unsurprisingly there was no mention of tablets in education at that stage
Cho et al. (2018)	The Effects of Using Mobile Devices on Student Achievement in Language Learning: A Meta‐Analysis	2018	Primary, postprimary, adult education	Mobile technology	Language learning	Focused specifically on the impact of technology on language learning, rather than wider educational outcomes. The proposed review will include English as an Additional Language if other inclusion criteria are met, as well as other literacy and numeracy interventions Limited search terms. Key words used: “language learning” AND achievement, AND “mobile” OR “m‐learning.” Grey literature search used Proquest Dissertations & Theses only. The proposed review will incorporate a more robust search process
Crompton et al. (2017)	The use of mobile learning in PK‐12 education: A systematic review.	2017	Grades PreK‐12 (4–18)	All mobile devices	This review considers the types of research carried out in the field of mobile learning in PK‐12, summarising the aims of the research, methodologies used, devices of interest, subject matters and educational levels considered	No analysis of effectiveness of interventions considered. Additionally, search strategy incorporates only the top 10 journals (as rated through Google Scholar Metrics) rather than a wider, exhaustive search strategy including grey literature. The proposed review will incorporate a more exhaustive search process
Harper (2018)	Technology and Teacher‐Student Interactions: A Review of Empirical Research	2018	Grades K‐12 or equivalent (5–18)	Wider technology	Impact of technology on teacher‐student interactions	Focused specifically on the impact that technology has on interactions of teachers and students in the classroom, rather than interventions where the device is used directly by pupils (as in proposed review) Additionally, the review included only published, peer reviewed studies
Herodotou (2018)	Young children and tablets: A systematic review of effects on learning and development.	2018	2–5	Tablets	Learning and development	Focused only on young children (pre‐school age). The proposed review would build on this information to focus on the next educational age group (4–11)
Langer (2018)	Rethinking mobile learning for development: Using the capability approach and a mixed‐methods systematic review to conceptualise the application of mobile technologies as an educational tool in low‐ and middle‐income countries	2018	All age groups	All mobile devices	The use of mobile technology to support educational development in low and middle‐income countries	Unable to access full text, however abstract plus connected papers shows the focus is on how mobile devices can support the specific educational challenges faced in low & middle income countries, for example engaging marginalised learners
Nguyen et al. (2015)	iPad in higher education—hype and hope	2015	Higher education (18 plus)	iPad (specifically, not including other tablet brands)	Wider learning outcomes	Only peer‐reviewed studies included Device is limited to branded i‐pads therefore narrowing the scope of the review. Older population considered than proposed review population (higher education)
Parsons (2014)	A mobile learning overview by timeline and mind map	2014	All age groups (including adults)	All mobile devices	This paper maps the ways in which mobile learning has been represented in the research field, including target audience (learner), locations (formal and informal education, everyday life, work‐based learning etc.), device and content.	No discussion in this review on the effectiveness of mobile devices on learning outcomes, rather presents a description of the focus taken by research
Sung et al. (2016)	The effects of integrating mobile devices with teaching and learning on students' learning performance: A meta‐analysis and research synthesis	2016	All age groups in formal education (including adults)	All mobile devices	Aims to determine the effectiveness of integrating mobile devices in education on student learning achievement, and potential moderating factors	This is similar in nature to the proposed study, however includes all age groups (including adults), all subject areas, and includes studies where technology is used both in and outside of the classroom. Search strategy only included peer reviewed journals. Searches included 1993–2013—a more up to date search is now needed. It will be useful to revisit this review following completion of the proposed review to identify any similarities or differences in findings. Proposed review will also seek to add detail to the discussion presented in this review
Talan (2020)	The Effect of Mobile Learning on Learning Performance: A Meta‐Analysis Study.	2020	All age groups in formal education (including adults)	All mobile devices	Thie study aims to determine the effect of mobile learning on students' learning performance, and consider moderating factors including education level, subject of study and duration	This review is close to the proposed review in terms of aims. However, it includes studies of mobile learning experience more widely rather than specifically within the classroom, including research focused on supporting engagement through learning at home. The review finds mobile learning to positively impact learning performance, however one reason posed is that “Mobile learning takes education out of the classroom.” Again, the proposed review will add to this learning rather than duplicate
Tingir et al. (2017)	Effects of mobile devices on K‐12 students' achievement: a meta‐analysis	2017	Grades K‐12 or equivalent (5–18)	All mobile devices	Science, maths and reading outcomes. Meta‐analyses were carried out across subject areas, device type and school type	Only peer‐reviewed studies included Geographical coverage is limited. While search criteria in relation to school class is not included, the majority of included studies are from countries which use the K‐12 system. Included articles are from the following countries: · United States (4) · Taiwan (5) · India · Turkey · Mexico · New Zealand · Spain (post‐primary study) Studies from the United Kingdom, Ireland, Australia, Canada and much of Europe are notably absent which represents a significant gap in terms of coverage
Torgerson et al. (2002)	A systematic review and meta‐analysis of the effectiveness of information and communication technology (ICT) on the teaching of spelling	2002	School age (4–18)	ICT in general	Teaching of spelling	Outdated—searches include research up to 2000 Focused only on the teaching of spelling, therefore applicability of evidence will be limited
Zhang and Nouri (2018)	A Systematic Review of Learning and Teaching with Tablets	2018	School age (4–18)	Tablets	Considers how pedagogical practices are supported by tablets in primary and secondary school	Does not consider learning outcomes specifically, rather focuses on teaching practice
Zucker et al. (2009)	The Effects of Electronic Books on Pre‐Kindergarten‐to‐Grade 5 Students' Literacy and Language Outcomes: A Research Synthesis	2009	Kindergarden to grade 5	e‐books	Literacy and language	Outdated. technology and practice have shifted significantly since 2009, in particular tablets which were introduced in 2009 Studied eBooks only, rather than wider technology

## APPENDIX C SAMPLE SEARCH STRATEGY: ERIC VIA EBSCO

**Table:**

S10	S3 AND S8 AND S9
S9	AB RCT OR “randomised control trial” OR “randomised controlled trial” OR “randomized control trial” OR “randomized controlled trial” OR randomised OR randomized OR placebo* OR (random* AND (allocat* OR assign*)OR (blind* AND (single OR double OR treble OR triple))
S8	S6 AND S7
S7	AB iPad OR tablet OR “tablet computer*” OR touchscreen OR app OR “handheld device” OR “handheld computer” OR “PDA” OR “personal digital assistant” OR “portable computer” OR '"e‐book” OR ebook OR “e‐reader” OR ereader OR “electronic storybook” OR “game* console” OR “digital game” OR smartphone OR “smart phone” OR “mobile phone” OR iPhone OR “cell* phone” OR “portable cell* phone” OR “mobile telephone” OR “cell* telephone” OR “transportable Cell* Phone” OR “1:1 comput*” OR “online instruction” OR “mobile learn*” OR M‐learning
S6	S4 OR S5
S5	AB numeracy OR number* OR math* OR arithmetic OR sums OR calculat* OR addition OR adding OR subtract* OR multiplication OR division OR count* OR algebra OR fractions OR decimal* OR geometr* OR statistic* OR “problem solving” OR “shape sort*” OR “mathematical literacy” OR “quantitative literacy”
S4	AB Literacy OR Reading OR Writing OR Handwriting OR Transcription OR “Verbal reasoning” OR “Critical thinking” OR Comprehension OR Notetaking OR Composition OR “Listening skills” OR Phonic* OR “phonemic awareness” OR Alphabet OR Spell* OR Vocabulary OR Punctuation OR Grammar OR “Reading fluency” OR “Speaking skills” OR “Spoken language” OR “Critical literacy” OR “literacy skills"
S3	S1 OR S2
S2	AB “Primary school” OR “Elementary school” OR “Junior school” OR Kindergarten OR “grade 1” OR “grade 2” OR “grade 3” OR “grade 4” OR “grade 5” OR “First Grade” OR “Second Grade” OR “Third Grade” OR “Fourth Grade” OR “Fifth Grade” OR “1st grade” OR “2nd grade” OR “3rd Grade” OR “4th Grade” OR “5th grade“
S1	AB (“Age* 4” OR “age* 5” OR “age* 6” OR “age* 7” or “age* 8” OR “age* 9” OR “age* 10” OR “age* 11” OR “4 year* old*” OR “5 year* old*” OR “6 year* old*” OR “7 year* old*” OR “8 year* old*” OR “9 year* old*” OR “10 year* old*” OR “11 year* old*” OR Child* OR boy OR girl) AND (school OR class* OR classroom)

The following tool has been adapted from guidelines set out in “Chapter 5: Collecting data” from the Cochrane Handbook for Systematic Reviews of Interventions. The tool will be piloted and refined before final data extraction.

## APPENDIX D DATA EXTRACTION FRAMEWORK

**Table:**

	Responses/notes (free text unless options given)	Location in text or source (e.g., pg, figure no.)
**General information**		
Name of data extractor:		
Date of data extraction		
Study ID or reference		
Report ID (if more than one report relating to the same study)		
If linked, IDs of linked reports		
Author/s		
Lead author contact details		
Year of publication		
Publication type (e.g., journal)	Journal article Technical report Dissertation/thesis Unpublished study Other (specify)	
Method of identification of source (e.g., via [xxx] database, hand‐search of journal, citation list of relevant article)		
Citation		
Study funder		
Is author affiliated with funder?	Y/N/Unclear	

Eligibility criteria		
	Responses/notes (free text unless options given)	Location in text or source (e.g., pg, figure no.)
Is an RCT used?	Y/N/Unclear	
Does the study consider use of mobile devices in the classroom to support delivery of mainstream curriculum?	Y/N/Unclear	
Are study participants in the correct age group (4–11) and within a primary school (or equivalent) class setting?	Y/N/Unclear	
Does the study focus on outcomes of interest? (literacy or numeracy and related skills)	Y/N/Unclear	
Do pupils use the device themselves (rather than the teacher)?	Y/N/Unclear	
Is study to be included?	Y/N/Unclear [if unclear, detail action to be taken]	
If excluded, confirm reason for exclusion	[insert text]	

Study methodology		
	Responses/notes (free text unless options given)	Location in text or source (e.g., pg, figure no.)
Aim of the study		
Study design (e.g., cluster randomised or RCT)		
Method of recruitment		
Sampling procedures		
Inclusion/exclusion criteria applied		
Unit of allocation (e.g., individual, class, school)		
Start date of study		
End date of study		
Duration of participation (from recruitment to last follow‐up)		

Participants:		
	Responses/notes (free text unless options given)	Location in text or source (e.g., pg, figure no.)
Population description		
Country/region of study		
Setting		
School FSM % (as a proxy for socioeconomic status)		
Method of recruitment of settings		
Inclusion criteria		
Exclusion criteria		
Total number of participants:		
‐ At the beginning		
‐ At the end		
‐ % participants who completed the study		
‐ Details of attrition at any stage of the study		
Age (SD, mean, range): ‐ For overall sample ‐ For each group reported		
Class/classes/grade reported	[list options]	
Are any children in sample below 4 or over 11?	Y/N/Unclear	
If yes, detail action taken to separate sample?		
Gender ‐ Of overall sample ‐ For each group	Report # and %	
Any other relevant characteristics specified?		
Was participation voluntary (for children)	Y/N/Unclear	
Consent received?	Y/N/Unclear	
If yes, who was consent sought from? (select all that apply)	Child Parent Teacher Other (specify)	
Method of consent (for each of the above)	Opt in Opt out	

Intervention:		
	Responses/notes (free text unless options given)	Location in text or source (e.g., pg, figure no.)
Name of the intervention		
Description of intervention (e.g., target outcome/s, key activities, delivery timing, duration & dosage (frequency, average length of each session (minutes), total number of sessions delivered))		
Technology device used	Tablet Smartphone Handheld game console Other (specify)	
Who owed the devices used?	School Pupils Combination Unclear	
How was the intervention accessed?	App downloaded to the device Website accessed online Hardware already on the device Other (please specify)	
Individual device use or groups?	Individual use In pairs Groups (3 or more) Unclear (specify)	
Cost of accessing the intervention (if available)		
How was the intervention undertaken?	Single activity Multiple activities Other (specify) Unclear (specify)	
Who delivered or supervised the intervention (e.g., teacher, classroom assistant)	Teacher Classroom assistant Other (specify)	
What was their role?		
Was specialist training required to deliver the intervention?	Y/N/Unclear (if yes, provide detail)	
What is the primary aim/target outcome of the intervention?		
What is/are the secondary aims/outcomes of the intervention?		
Number of intervention groups		
Number of control groups		
Any differences between the groups?		
Provide details of the control/comparison condition	Business as usual Other intervention Waiting list Other?	
Were any other interventions being delivered concurrently which may have impacted outcomes?	Y/N/unclear	
If yes, provide detail		
Was effort made to assess fidelity?	Y/N/Unclear	
If yes, provide detail		

Assessment of level of intervention on the SAMR model: (data extractor/s will have further details of SAMR framework and examples available on which to make judgement)		
	Responses/notes (free text unless options given)	Location in text or source (e.g., pg, figure no.)
Substitution: technology replaces a traditional activity but activity remains fundamentally the same		
Augmentation: technology replaces the traditional activity and adds some additional function		
Modification: Technology significantly alters the original task		
Redefinition: technology allows task to be redefined to include previously unachievable activities		

Outcomes		
	Responses/notes (free text unless options given)	Location in text or source (e.g., pg, figure no.)
Primary outcomes assessed	Academic achievement: Literacy (specify) Numeracy (specify) Other (specify) Unclear (specify)	
Answer the following questions for each primary outcome assessed:		
Measurement tools used and details of administration		
Unit of measurement (if relevant)		
Upper and lower limit of measurement (& details of meaning, e.g., high score = good)		
Were tools validated?	Y/N/unclear (provide detail)	
Time points at which measures were assessed	After intervention only Before and after intervention Unclear (specify)	
How long after intervention was data collected?	Between 0 and 3 months Between 4 and 6 months Between 6 and 9 months Longer than 9 months (specify) Unclear (specify)	
Was follow up data collected?	Yes (specify time points) No Unclear	
Summary data collected		
Missing data?	Y/N/unclear Provide detail	
Action taken to access summary data (if not provided)	(if no above)	
Statistical analyses carried out		
Assumed risk estimate		
Power (e.g., power & sample size calculation, level of power achieved		

Findings		
	Responses/notes (free text unless options given)	Location in text or source (e.g., pg, figure no.)
Key conclusions of authors		
References to other relevant studies identified:		
Details on further correspondence needed: (who with, what information is needed, when/how will it be requested)		

(repeat this table for each outcome)

**Table:**

Data collection and analysis				
Comparison	
Outcome				
Type of outcome (e.g., dichotomous, continuous)	
Subgroup				
Time point *(specify from start or end of intervention)*	
Post‐intervention or change from baseline? (if continuous)				
No of participants	Intervention		Control/comparison	
Results (if dichotomous)	Intervention		Comparison	
	No. with event	Total in group	No. with event	Total in group
Results (if continuous)	Intervention		Control	
	Intervention result	SE (or other variance)	Control result	SE (or other variance)
	Overall results		SE (or other variance)	
Any other results reported *(e.g., odds ratio, risk difference, CI or P value)*				
No. missing participants		
Reasons missing				
No. participants moved from other group		
Reasons moved		
Unit of analysis *(by individuals, cluster/groups)*				
Statistical methods used and appropriateness of these *(e.g., adjustment for correlation)*	
Reanalysis required? *(specify, e.g., correlation adjustment)*	Y/N/unclear (specify)			
Reanalysis possible?	Y/N/unclear (specify)			
Reanalysed results				
